# Manganese Sulfate Nanocomposites Fabricated by Hot-Melt Extrusion for Chemodynamic Therapy of Colorectal Cancer

**DOI:** 10.3390/pharmaceutics15071831

**Published:** 2023-06-27

**Authors:** Da In Jeong, Sungyun Kim, Ja Seong Koo, Song Yi Lee, Minju Kim, Kwang Yeol Kim, Md Obyedul Kalam Azad, Mrinmoy Karmakar, Seongnam Chu, Byung-Jo Chae, Wie-Soo Kang, Hyun-Jong Cho

**Affiliations:** 1 College of Pharmacy, Kangwon National University, Chuncheon 24341, Republic of Korea; jdi0327@kangwon.ac.kr (D.I.J.); sungyun@kangwon.ac.kr (S.K.); pharmchu@dhpharm.co.kr (S.C.); 2 Department of Animal Resources Science, College of Animal Life Sciences, Kangwon National University, Chuncheon 24341, Republic of Korea; 3 School of Animal Life Convergence Science, Hankyong National University, Anseong 17579, Republic of Korea; 4 Institute of Applied Humanimal Science, Hankyong National University, Anseong 17579, Republic of Korea; 5 Darby Genetics Inc., Anseong 17529, Republic of Korea; 6 Department of Bio-Health Technology, College of Biomedical Science, Kangwon National University, Chuncheon 24341, Republic of Korea; 7 Department of Chemistry and Biochemistry, Food and Dairy Innovation Center, Boise State University, Boise, ID 83725, USA; 8 Daehwa Pharmaceutical Co., Ltd., Seoul 06699, Republic of Korea

**Keywords:** chemodynamic therapy, Fenton-like reaction, hot-melt extrusion, manganese sulfate, nanocomposite

## Abstract

The development of metal salts-based nanocomposites is highly desired for the Fenton or Fenton-like reaction-based chemodynamic therapy of cancer. Manganese sulfate (MnSO_4_)-dispersed nanoparticles (NPs) were fabricated with a hot-melt extrusion (HME) system for the chemodynamic therapy of colorectal cancer in this study. MnSO_4_ was homogeneously distributed in polyethylene glycol (PEG) 6000 (as a hydrophilic polymer) with the aid of surfactants (Span 80 and Tween 80) by HME processing. Nano-size distribution was achieved after dispersing the pulverized extrudate of MnSO_4_-based composite in the aqueous media. The distribution of MnSO_4_ in HME extrudate and the interactions between MnSO_4_ and pharmaceutical additives were elucidated by Fourier-transform infrared, X-ray diffractometry, X-ray photoelectron spectroscopy, and scanning electron microscopy analyses. Hydroxyl radical generation efficiency by the Fenton-like chemistry capability of Mn^2+^ ion was also confirmed by catalytic assays. By using the intrinsic H_2_O_2_ in cancer cells, MnSO_4_ NPs provided an elevated cellular reactive oxygen species level, apoptosis induction capability, and antiproliferation efficiency. The designed HME-processed MnSO_4_ formulation can be efficiently used for the chemodynamic therapy of colorectal cancer.

## 1. Introduction

Hot-melt extrusion (HME) has been widely engaged for preparing drug formulations due to its continuous process, cost-effectiveness, large scale-up production, no downstream process, high throughput characteristic, solvent-free property, and unit operation [1,2,3]. Several machine and operation factors, such as screw type (e.g., single, twin, and multi screws), screw design, screw speed, feed rate, barrel temperature, and die shape, may govern the physicochemical properties of extrudates [1]. Additionally, physical and chemical features of active pharmaceutical ingredients, carriers, and plasticizers should be considered for the optimization of HME process [1]. In the commercial market, several products have been supplied as shaped (rod or ring) systems and amorphous or crystalline dispersion for ophthalmic inserts, implants, devices, and oral formulations [2]. HME-processed products may have the following outcomes: solubility/bioavailability improvement, taste masking, and controlled/targeted drug release [2]. Various types of pharmaceutical dosage forms, such as films, granules, multicomponent systems (e.g., salts, co-amorphous systems, and co-crystal systems), nanoparticles, pellet, self-microemulsifying drug delivery systems, semi-solid products (e.g., creams, gels, and ointments), and solid implants, can be prepared by the HME technique [3]. These days, an HME system has been coupled with other downstream equipment (e.g., high-pressure homogenizer, pelletizer, and 3D printer) for producing advanced formulations [3].

In cases of organic small chemicals, HME has been selected for improving aqueous solubility, dissolution, and absorption, controlling drug release, and masking bitter tastes [4,5,6,7]. However, there were few reports regarding the fabrication of delivery systems for inorganic substances by HME technique [8,9,10]. In our previous studies [8,9,10], hydrophilic polymer and surfactants have been introduced to make colloidal systems of mineral salts (e.g., ZnSO_4_, FeSO_4_, and CuSO_4_) after dispersing in aqueous media. Herein, polyethylene glycol (PEG) 6000 was used as a hydrophilic polymer and Span 80 and Tween 80 were added as non-ionic surfactants for the homogeneous dispersion of MnSO_4_. PEG 6000 can act as a polymer matrix for the distribution of MnSO_4_ and both Span 80 and Tween 80 can reduce the surface tension during the particle formation process in the aqueous media. The combination of PEG 6000, Span 80, and Tween 80 has been successfully applied to make colloidal systems of ZnSO_4_, FeSO_4_, and CuSO_4_ [8,9,10]. Although the same feeding ratio of PEG 6000, Span 80, and Tween 80 has been used for HME processing, those metal salts (e.g., ZnSO_4_, FeSO_4_, and CuSO_4_) in our previous studies have different physicochemical properties (i.e., solubility and dispersibility) compared with MnSO_4_. HME processing and formulation design were successfully applied to a MnSO_4_ molecule in this study. Those HME-processed mineral salts-based particles exhibited nano-sized structures following dispersion in aqueous media. Nano-sized particulate systems may generally possess several advantages such as specific delivery to the target region, the improvement of drug safety and efficacy, and controlled drug cargo release, as reported [11]. This study may have critical implications in the fabrication of MnSO_4_-based nanoparticles (NPs) with polymer and surfactants by HME processing.

Moreover, HME-processed MnSO_4_ NPs were applied to the chemodynamic therapy of colorectal cancer in this investigation. Cancer cells may have higher H_2_O_2_ levels compared to normal cells; therefore, a H_2_O_2_-responsive system may possess cancer cell targeting capability [12]. Mn^2+^ ion, usually provided by MnO_2_, can provide a Fenton-like reaction; therefore, it can generate hydroxyl radical from cellular H_2_O_2_ [13,14,15]. In the current study, MnSO_4_ has been used as the source of Mn^2+^ ion, and the HME process was introduced to make nano-size structures for enhanced entry to cancer cells. The generation of toxic hydroxyl radicals may inhibit the proliferation of cancer cells through the elevation of the cellular reactive oxygen species (ROS) level and the induction of other cell death mechanisms (e.g., apoptosis). It is expected that the developed MnSO_4_ NPs may be efficient chemodynamic therapeutic agents for colorectal cancer.

## 2. Materials and Methods

### 2.1. Materials

MnSO_4_ (MnSO_4_·H_2_O) was acquired from TMC Co., Ltd. (Anyang, Republic of Korea). PEG 6000 was provided by Samchun Pure Chemical Co., Ltd. (Pyeongtaek, Republic of Korea). Span 80 and Tween 80 were purchased from Daejung Chemical & Metals Co., Ltd. (Siheung, Republic of Korea). 2′,7′-Dichlorofluorescin diacetate (DCFH-DA), 3,3′,5,5′-tetramethylbenzidine dihydrochloride hydrate (TMB DH), and methylene blue (MB) were purchased from Sigma–Aldrich (Saint Louis, MO, USA). Dulbecco’s modified Eagle’s medium (DMEM), penicillin-streptomycin, and fetal bovine serum (FBS) were obtained from Gibco Life Technologies, Inc. (Grand Island, NY, USA).

### 2.2. Production and Particle Characterization of MnSO_4_ Extrudate-Based Nanocomposites

#### 2.2.1. Production of MnSO_4_ Formulations by HME

MnSO_4_, PEG 6000, Span 80, and Tween 80 were blended at a 20:64:12:4 ratio (*w*/*w*/*w*/*w*) prior to putting those materials into the extruder [8,9,10,16]. Those blended materials were translocated to the hopper part of HME (45 g/min rate). A hot-melt extruder installed with twin-screw (STS-25HS, Hankook E.M. Ltd., Pyeongtaek, Republic of Korea) and a round-shaped die (1 mm diameter) was utilized for the fabrication of MnSO_4_-based extrudates. Temperatures in the barrel and die parts were set as 45 °C and 40 °C, respectively. The screw speed was maintained at 150 rpm in this HME step. After moving through the conveying and kneading parts in the barrel, specimens were extruded from the die part. Extruded materials were hardened and pulverized by the grinder (HBL-3500S, Samyang Electronics Co., Gunpo, Republic of Korea).

#### 2.2.2. Particle Characterization of MnSO_4_ NPs

The mean diameter of the MnSO_4_ powder was determined by laser diffraction particle size analysis. MnSO_4_ was dispersed in ethanol and the size-dependent volume density profile was obtained by a laser diffraction particle size analyzer (Mastersizer 3000, Malvern Instruments Ltd., Malvern, UK) [17].

The particle properties of MnSO_4_ NPs in distilled water (DW) were investigated. Hydrodynamic diameter, polydispersity index, and zeta potential values of MnSO_4_ NPs dispersed in DW (at 10 mg/mL) were measured by dynamic light scattering and laser Doppler methods (ELS-Z1000; Otsuka Electronics, Tokyo, Japan) [8,9,10].

The morphology of MnSO_4_ NPs (in DW) was observed by transmission electron microscopy (TEM). MnSO_4_ NPs (10 mg/mL in DW) were put onto the copper grid with film, the sample was stained with uranyl acetate (1%), and the liquid content was dried in an air stream for 10 min. The particle shape was observed by TEM (CM120; Philips, Amsterdam, The Netherlands).

The content of Mn in MnSO_4_ NPs was determined by inductively coupled plasma-optical emission spectrometry (ICP-OES; Optima 7300 DV, PerkinElmer, Inc., Waltham, MA, USA). Before ICP-OES analysis, the specimen was dissolved in nitric acid.

Incubation time-dependent mean diameter and size distribution features of MnSO_4_ NPs were determined by a dynamic light scattering method (ELS-Z1000, Otsuka Electronics). MnSO_4_ NPs (at 10 mg/mL) were dispersed in phosphate buffered saline (PBS, pH 7.4) or FBS solution (50%, *v*/*v*) and they were incubated for 24 h. After incubating for 0.5, 1, 3, 6, and 24 h, hydrodynamic diameter and polydispersity index values were measured.

### 2.3. Solid-State Studies

#### 2.3.1. Fourier-Transform Infrared (FT-IR) Spectroscopy Analysis

Alteration in the chemical functions of MnSO_4_, freeze-dried mixture (MnSO_4_, PEG 6000, Span 80, and Tween 80), and MnSO_4_ NPs was studied by FT-IR analysis. Transmittance (%) values of MnSO_4_, the freeze-dried mixture, and MnSO_4_ NPs were scanned using a Frontier FT-IR spectrometer (PerkinElmer Inc., Buckinghamshire, UK). FT-IR spectra were obtained in the attenuated total reflectance mode. Transmittance (%) values of MnSO_4_, the freeze-dried mixture, and MnSO_4_ NPs were monitored in a 400–4000 cm^−1^ wavenumber range.

#### 2.3.2. X-ray Diffractometry (XRD) Assay

The crystal properties of MnSO_4_, the freeze-dried mixture, and MnSO_4_ NPs were explored by a Philips X′Pert PRO MPD diffractometer (PANalytical Corp., Almero, The Netherlands). The intensity values according to angle (2θ) at the 5–80° range were measured. The generator voltage and tube current were set as 40 kV and 30 mA, respectively. The scan time per step and scan step size were 8.67 s and 0.013°, respectively.

#### 2.3.3. X-ray Photoelectron Spectroscopy (XPS) Study

The elemental composition of MnSO_4_ and MnSO_4_ NPs was determined by an XPS (K-Alpha+, Thermo Fisher Scientific, East Grinstead, UK) system. The atomic contents (%) in MnSO_4_ (Mn 2p, O 1s, Ca 2p, C 1s, and S 2p) and MnSO_4_ NPs (Mn 2p, O 1s, C 1s, and S 2p) were measured. Al K_α_ X-ray (as a source gun) was used in this test. Peak patterns of C 1s, O 1s, Mn 2p, and S 2p were analyzed in a narrow binding energy range.

#### 2.3.4. Field Emission Scanning Electron Microscopy (FE-SEM)/Energy Dispersive Spectrometry (EDS) Study

FE-SEM (JSM-7900F, JEOL, Tokyo, Japan) combined with EDS was applied to identify the distribution of elements on MnSO_4_ and MnSO_4_ NPs. Atomic contents of C, O, S, and Mn in MnSO_4_ and MnSO_4_ NPs were quantitatively determined.

### 2.4. Catalytic Assays

#### 2.4.1. 3,3′,5,5′- Tetramethylbenzidine (TMB) Assay

The hydroxyl radical generation activity was examined by a TMB-based assay [18]. Each specimen (0.2 mL) of MnSO_4_ and MnSO_4_ NPs (at 1000 μg/mL MnSO_4_ concentration) was put into the microtube and 0.03% H_2_O_2_ solution (0.2 mL) was added. Then, 10 mM TMB DH (0.4 mL) was added to each sample and they were incubated at 37 °C for reaction. The reactant (0.2 mL) was acquired at 0, 10, 20, and 30 min and the corresponding absorbance was detected at 650 nm by a microplate reader (SpectraMax i3, Molecular Devices, Sunnyvale, CA, USA).

#### 2.4.2. MB Assay

The hydroxyl radical scavenging effect was studied by an MB-based assay [19]. Each specimen (0.5 mL) of MnSO_4_ and MnSO_4_ NPs (at 1000 μg/mL MnSO_4_ concentration) was put into a dialysis tube (molecular weight cut-off: 12–14 kDa). An H_2_O_2_ solution (0.03%, 2.5 mL) was added to that sample and they were incubated at 37 °C for 30 min. Subsequently, an MB solution (20 μg/mL) was added to that mixture and the reactant (0.2 mL) was collected at 0, 15, 30, 60, and 120 min. The absorbance at 650 nm was measured by a microplate reader.

### 2.5. In Vitro Anticancer Activity Tests

#### 2.5.1. Antiproliferation Assay

The antiproliferation potential of MnSO_4_ and MnSO_4_ NPs was assessed in CT-26 (colon carcinoma) cells with a colorimetric assay. CT-26 cells were obtained from the Korean Cell Line Bank (Seoul, Republic of Korea). Those cells were cultured in DMEM containing FBS (10%, *v*/*v*) and penicillin-streptomycin (1%, *v*/*v*) at 37 °C. CT-26 cells (at 5.0 × 10^3^ cells per well) were seeded in 96-well plate and they were incubated at 37 °C for 24 h. MnSO_4_ and MnSO_4_ NPs (at 10, 25, 50, 100, and 200 μg/mL MnSO_4_ concentrations) were applied to the cells and they were incubated for 72 h. After eliminating each sample, CellTiter 96^®^ AQueous One Solution Cell Proliferation Assay Reagent (Promega Corp., Fitchburg, WI, USA) was applied to the cells and incubated at 37 °C. The absorbance at 490 nm was detected by a microplate reader.

#### 2.5.2. Cellular ROS Assay

CT-26 cells were added to a 6-well plate at 1.0 × 10^5^ cells/well density. The next day, each specimen of MnSO_4_ and MnSO_4_ NPs (at 200 μg/mL MnSO_4_ concentration) was treated to the cells and they were incubated for 24 h. The cells were washed with cold PBS two times and stained by DCFH-DA (10 μM) for 20 min at 37 °C. Then, the cells were resuspended with FBS solution (2%, *v*/*v*). The fluorescence intensity of cellular ROS was measured by flow cytometry (BD Bioscience, San Diego, CA, USA).

#### 2.5.3. Apoptosis Assay

CT-26 cells were seeded in a 6-well plate at a density of 1.0 × 10^5^ cells per well. Each specimen of MnSO_4_ and MnSO_4_ NPs (at 200 μg/mL MnSO_4_ concentration) was treated to the cells and incubated for 24 h. The cells were washed with cold PBS two times and resuspended in the binding buffer at a concentration of 1.0 × 10^6^ cells/mL. The cells were stained with FITC Annexin V and propidium iodide (PI) in the binding buffer and analyzed by flow cytometry.

### 2.6. Data Analysis

Statistical analyses of data were performed with a two-tailed *t*-test and analysis of variance. Each experiment was repeated at least three times. Data are provided as the mean ± standard deviation (SD).

## 3. Results and Discussion

### 3.1. Fabrication and Particle Property Tests of MnSO_4_ NPs

**Figure 1:**
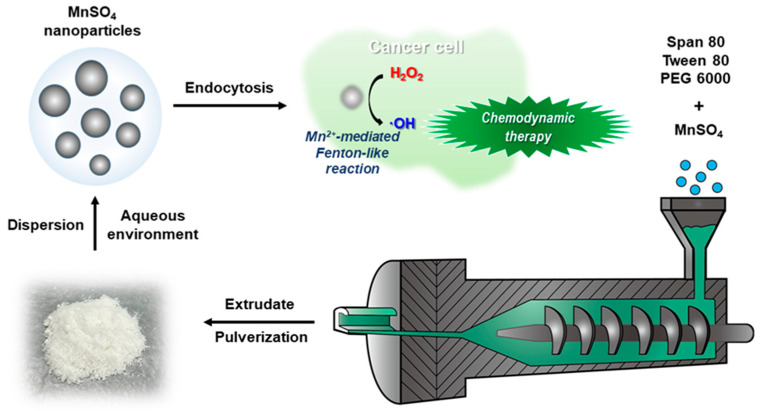
Schematic illustration of MnSO_4_ NPs production by HME process.

MnSO_4_ NPs were fabricated by the HME process for the chemodynamic therapy of cancer in this study (Figure 1). MnSO_4_ was homogeneously dispersed in hydrophilic polymer (PEG 6000) and non-ionic surfactants (Span 80 and Tween 80) by a twin-screw-based HME system. As observed in our previous reports [8,9], the combination of PEG 6000, Span 80, and Tween 80 was successfully engaged to prepare nano-sized particles of FeSO_4_ or CuSO_4_ following their dispersion in the aqueous environment. MnSO_4_/PEG 6000/Span 80/Tween 80 extrudate was acquired by the HME process and was pulverized for the convenient preparation of nano-size vesicles after dispersing in the aqueous media. Nano-sized particles of MnSO_4_ may be easily introduced to cancer cells [20] and they may attribute to the conversion of intrinsic H_2_O_2_ to hydroxyl radical by a Fenton-like reaction of Mn^2+^ ion.

**Figure 2:**
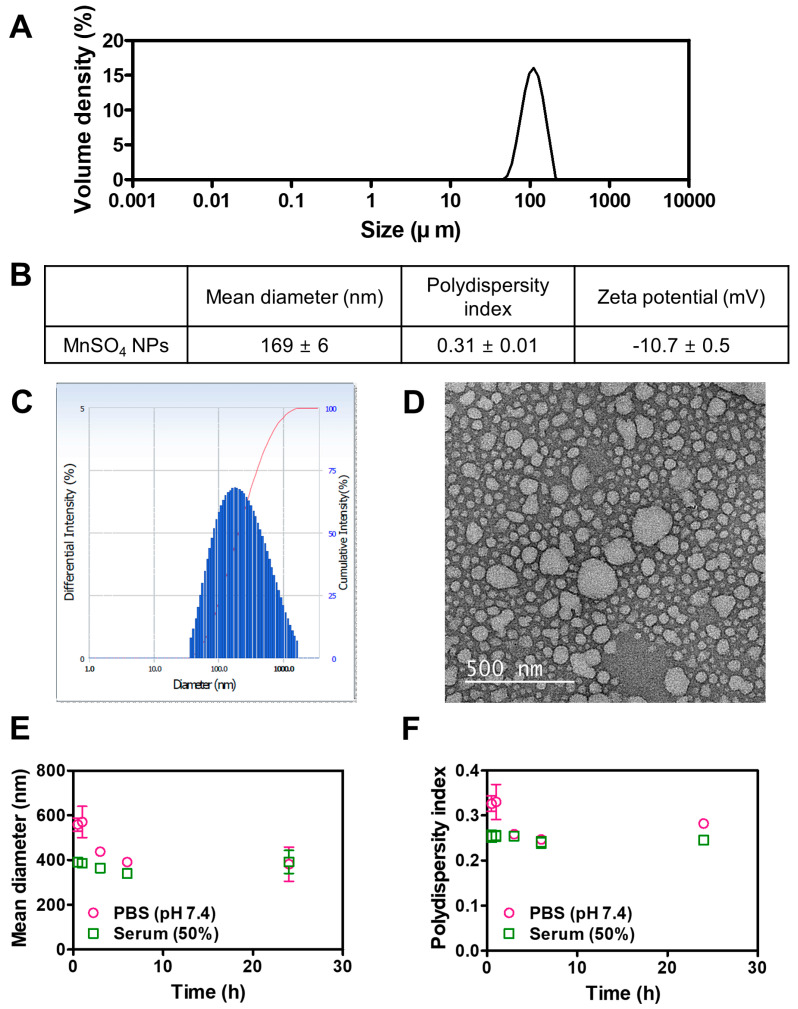
Particle characteristics of HME-processed MnSO_4_ NPs. (**A**) Particle size distribution of MnSO_4_. (**B**) Mean diameter, polydispersity index, and zeta potential values of MnSO_4_ NPs. Mean diameter and polydispersity index values were calculated by measuring 50 cycles per each sample. Average and deviation data were acquired with three batches of sample. Each point represents mean ± SD (*n* = 3). (**C**) Particle size distribution of MnSO_4_ NPs dispersed in DW. (**D**) TEM image of MnSO_4_ NPs dispersed in DW. Scale bar length: 500 nm. (**E**) Incubation time-dependent mean diameter profiles in PBS (pH 7.4) and serum with DW (50%, *v*/*v*). Each point represents mean ± SD (*n* = 3). (**F**) Incubation time-dependent polydispersity index profiles in PBS (pH 7.4) and serum with DW (50%, *v*/*v*). Each point represents mean ± SD (*n* = 3).

The particle features of MnSO_4_ NPs produced by the HME technique were explored as shown in Figure 2. The diameter of MnSO_4_ dispersed in ethanol was determined by a particle size analyzer (Figure 2A). The D_v_ (10), D_v_ (50), and D_v_ (90) values of MnSO_4_ were 78, 116, and 169 μm, respectively. The micron size of the MnSO_4_ powder was confirmed by particle size analysis.

The particle properties of HME-processed MnSO_4_ composites (MnSO_4_ NPs) were explored by size, distribution, zeta potential, encapsulation efficiency, and morphology analyses (Figure 2B–F). The hydrodynamic diameter of MnSO_4_ NPs in DW was 169 nm, and that group exhibited unimodal size distribution (Figure 2B,C). MnSO_4_ seems to be successfully dispersed in the hydrophilic polymer (PEG 6000) and surfactants (Span 80 and Tween 80), reducing the surface tension of particles. By the aid of twin-screw-enabled HME processing, MnSO_4_ seems to be homogeneously dispersed in the polymer matrix and it produced nano-sized particles in the aqueous media. Therefore, a significant size reduction effect from micron (MnSO_4_ powder) to nano (MnSO_4_ NPs) size was accomplished with the HME process with the hydrophilic polymer and surfactants. The zeta potential value of MnSO_4_ NPs was −10.7 mV and spherical in shape, with the corresponding diameter, was also shown in the TEM image (Figure 2B,D). The encapsulation efficiency of MnSO_4_ in MnSO_4_ NPs was around 100%, indicating the successful encapsulation of MnSO_4_ in polymer/surfactant-based particles.

The particle stability in the biological matrices was evaluated by particle size analysis in aqueous buffer and serum media (Figure 2E,F). In both PBS (pH 7.4) and serum solution (50% FBS), the initial hydrodynamic diameters of MnSO_4_ NPs were higher than that in DW (Figure 2B,E). This may be due to the interactions between MnSO_4_ NPs and buffer salts in PBS or proteins in serum media. Nevertheless, the hydrodynamic diameters of MnSO_4_ NPs in PBS (pH 7.4) and serum media (50% FBS) after 24 h incubation were 381 and 392 nm, respectively. The polydispersity index values of MnSO_4_ NPs in PBS (pH 7.4) and serum media (50% FBS) after 24 h incubation were 0.28 and 0.25, respectively. In particular, the existence of the PEG layer in MnSO_4_ NPs may inhibit the opsonization (which can lead to early elimination by mononuclear phagocyte system) and attribute to the maintenance of individual particles without the formation of aggregation [21,22]. Those results mean the maintenance of the nano-size even after incubation in the aqueous buffer or serum conditions, simulating the biological fluids. This may imply the safe application of developed MnSO_4_ NPs for cancer treatment.

### 3.2. Solid-State Features of MnSO_4_ NPs

**Figure 3:**
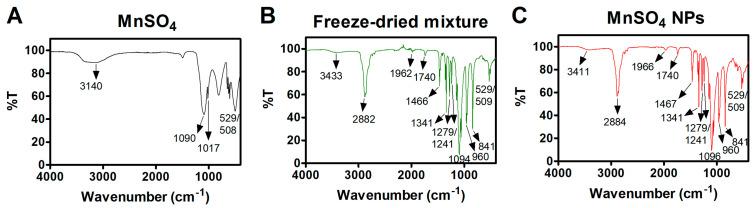
FT-IR spectra of (**A**) MnSO_4_, (**B**) freeze-dried mixture, and (**C**) MnSO_4_ NPs. Wavenumber-dependent transmittance value (%T) is plotted.

**Figure 4:**
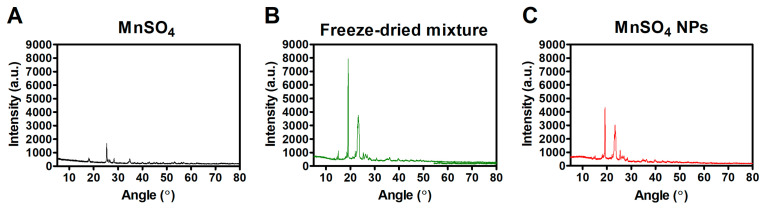
XRD patterns of (**A**) MnSO_4_, (**B**) freeze-dried mixture, and (**C**) MnSO_4_ NPs.

**Figure 5:**
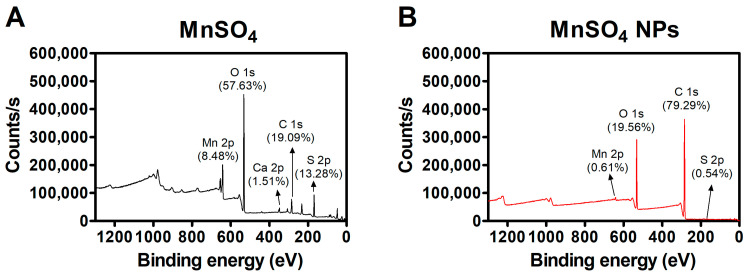
XPS data of (**A**) MnSO_4_ and (**B**) MnSO_4_ NPs.

**Figure 6:**
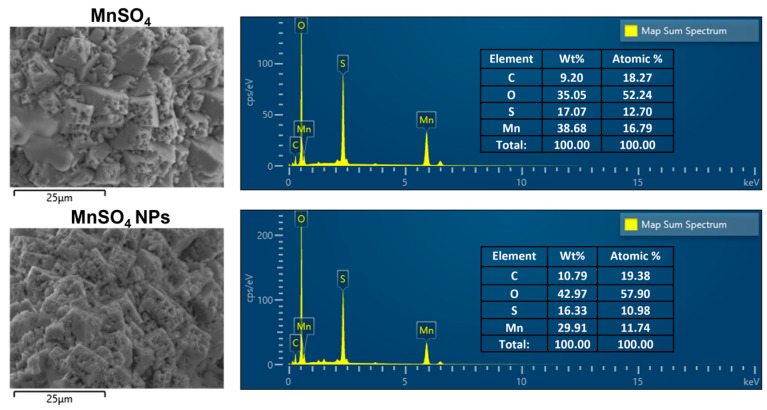
SEM images and EDS analysis data of MnSO_4_ and MnSO_4_ NPs. Weight portion (Wt%) and atomic contents (Atomic %) are included in the inset.

The solid-state features of fabricated MnSO_4_ NPs by HME process were explored by FT-IR, XRD, XPS, and SEM-EDS tests (Figure 3, Figure 4, Figure 5 and Figure 6 and Figures S1–S4, Tables S1 and S2). The existence of MnSO_4_ and its interactions with pharmaceutical additives were elucidated by FT-IR analysis (Figure 3 and Table S1). MnSO_4_ was characterized by several shifts, such as *v*_1_SO_4_^2−^ (1017 cm^−1^), *v*_3_SO_4_^2−^ (1090 cm^−1^), and the O–H stretching band (3140 cm^−1^) (Figure 3A) [23]. Those peaks were also observed at 960 cm^−1^, 1094 cm^−1^, and 3433 cm^−1^, respectively, in the freeze-dried mixture group (Figure 3B). MnSO_4_ in MnSO_4_ NPs was also featured by *v*_1_SO_4_^2−^ (960 cm^−1^), *v*_3_SO_4_^2−^ (1096 cm^−1^), and the O–H stretching band (3411 cm^−1^) (Figure 3C). The FT-IR data of the freeze-dried mixture and MnSO_4_ NPs groups were almost similar, indicating similar interactions between MnSO_4_ and pharmaceutical excipients during freeze-drying and the HME process (Table S1). The unaltered doublet Mn–O peaks of MnSO_4_ (529/508 cm^−1^) in MnSO_4_ NPs (529/509 cm^−1^) envisaged the incorporation of MnSO_4_ in MnSO_4_ NPs [24,25,26]. In particular, peaks at 1740 cm^−1^ and 2884/1467 cm^−1^ were attributed to the C=O stretching of ester functionality of Span 80 and Tween 80 and the C–H stretching/deformation bands of –CH_2_– of Span 80, Tween 80, and PEG 6000, respectively [27]. The appearance of alkane- and ester-specific peaks in the FT-IR spectra of MnSO_4_ NPs indicated the successful formation of MnSO_4_-based nanovesicles by the HME process.

The crystalline feature of MnSO_4_ included in NPs was explored by XRD assay (Figure 4). Representative peaks were shown at 18.04°, 25.38°, 28.37°, and 34.81° in the spectrum of MnSO_4_ [28]. Characteristic shifts were observed at 19.23°, 23.34°, and 25.42° in the profile of the MnSO_4_ NPs group. Those peaks in MnSO*_4_* NPs group were very similar to those in freeze-dried mixture group with attenuated intensity. The HME process can likely be attributed to less crystallization of the ingredients compared to freeze-drying. The existence of PEG 6000, Span 80, and Tween 80 and their interactions with MnSO_4_ were observed in the XRD profile of the MnSO_4_ NPs group.

The atomic distribution in the outer surface of prepared specimens was investigated with an XPS assay (Figure 5 and Figure S1–S4, Table S2). The contents of Mn 2p, S 2p, and O 1s in the MnSO_4_ group were 8.48%, 13.28%, and 57.63%, respectively. However, in the MnSO_4_ NPs group, the values of Mn 2p, S 2p, and O 1s were changed to 0.61%, 0.54%, and 19.56%, respectively. HME processing with pharmaceutical excipients seems to alter the atomic distribution in fabricated MnSO_4_ NPs. The MnSO_4_ molecule might move to the inner part of the NP sample according to the data of XPS analysis. A dramatic change in C 1s and O 1s values between MnSO_4_ and MnSO_4_ NPs groups may be due to the existence of PEG 6000, Span 80, and Tween 80 in the tested region.

The incorporation of PEG 6000, Span 80, and Tween 80 could also be inferred from the deconvoluted C 1s and O 1s spectrum of MnSO_4_ NPs. The deconvoluted C 1s peaks were observed at 284.53, 286.05, and 288.70 eV, attributed to –***C***H_2_–/–***C***H=***C***H–, >***C***H–OH/–***C***H_2_–OH, and –(***C***=O)–O–, respectively (Figure S1 and Table S2) [29]. Moreover, two distinct peaks were detected at 532.35 and 533.22 eV in the deconvoluted O 1s spectrum of MnSO_4_ NPs assigned to –(C=***O***)–O– and >CH–***O***H/–CH_2_–***O***H (Figure S2B) [30].

The peaks at 654.55 and 642.01 eV attributed to Mn 2p_1/2_ and Mn 2p_3/2_ in Mn 2p spectrum of MnSO_4_ [31] shifted to 654.29 and 641.59 eV, respectively, in the Mn 2p spectrum of MnSO_4_ NPs (Figure S3). Such a relatively lesser negative shift (i.e., –0.26 and –0.42 eV, respectively) of Mn 2p peaks implied the prevalence of weak ionic interaction between Mn(II) of MnSO_4_ and –(C=O)–O–/>CH–OH/–CH_2_–OH of PEG 6000, Span 80, and Tween 80 [32]. It was further confirmed by the shift of O 1s and S 2p peaks of SO_4_^2−^ from 531.68 and 168.29/169.33 eV ) of MnSO_4_ to 531.57 and 164.65/168.75 eV (Figures S2B and S4B) in MnSO_4_ NPs, respectively.

Atomic localization was also investigated with SEM equipped with an EDS technique (Figure 6). The atomic percentage values of Mn and S in the MnSO_4_ group were 16.79% and 12.70%, respectively. They were clearly changed to 11.74% (Mn) and 10.98% (S) in the MnSO_4_ NPs group. The alteration of tested atoms in the MnSO_4_ NPs group compared to the MnSO_4_ group indicates the formation of nano-sized particles composed of MnSO_4_, PEG 6000, Span 80, and Tween 80 by the HME process. All of these findings revealed in the solid-state studies suggest the successful fabrication of MnSO_4_-based nanovesicles following dispersion in the aqueous media.

### 3.3. Catalytic Features

**Figure 7:**
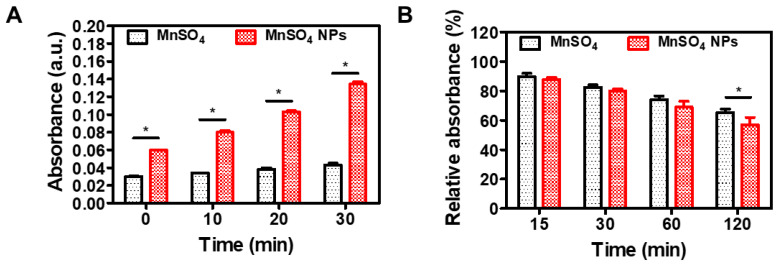
Catalytic assays of MnSO_4_ and MnSO_4_ NPs. (**A**) TMB assay data of MnSO_4_ and MnSO_4_ NPs. Each point represents mean ± SD (*n* = 3). * *p* < 0.05, between two groups. (**B**) MB assay data of MnSO_4_ and MnSO_4_ NPs. Each point represents mean ± SD (*n* = 3). * *p* < 0.05, between two groups.

The catalytic activity of developed MnSO_4_ NPs was investigated with TMB and MB assays (Figure 7). Mn^2+^ ion (from MnSO_4_) can degrade cellular H_2_O_2_ to OH radical via a Fenton-like reaction [33]. The H_2_O_2_ level in the tumor cell is clearly higher than that in normal cells; therefore, this H_2_O_2_-responsive catalytic vehicle may possess tumor-selective therapeutic effects. OH radical may oxidize TMB (colorless) to its blue product; thus, its absorbance at the 650 nm wavelength can be used for the assessment of OH radical generation [34]. In this study (Figure 7A), there was an obvious difference between MnSO_4_ and MnSO_4_ NPs groups (*p* < 0.05). Of note, an increasing pattern of the absorbance value was observed in the MnSO_4_ NPs group rather than the MnSO_4_ group. Compared to micron-sized MnSO_4_ powder, MnSO_4_ NPs may possess large surface area in the aqueous media. This may result in elevated Fenton-like reaction-based conversion of H_2_O_2_ to OH radical.

The OH radical generation efficiency of designed MnSO_4_ NPs was also assessed by MB-based assay (Figure 7B). It is known that OH radical may attack the C–S^+^=C group as the first step of MB degradation and the degradation of the aromatic ring will make the MB solution transparent [35]. The MB degradation rate can be used for the evaluation of OH radical production [36]. As shown in Figure 7B, both MnSO_4_ and MnSO_4_ NPs groups had decreasing profiles of MB for 120 min incubation. Interestingly, there was a significant difference between MnSO_4_ and MnSO_4_ NPs groups at 120 min (*p* < 0.05). An approximately 43% reduction in the absorbance value of MB in the MnSO_4_ NPs group may imply the continuous production of OH radical during the tested reaction process. All of these data from catalytic assays suggest the successful generation of hydroxyl radicals aiming at the chemodynamic therapy of cancer.

### 3.4. In Vitro Anticancer Activities

**Figure 8:**
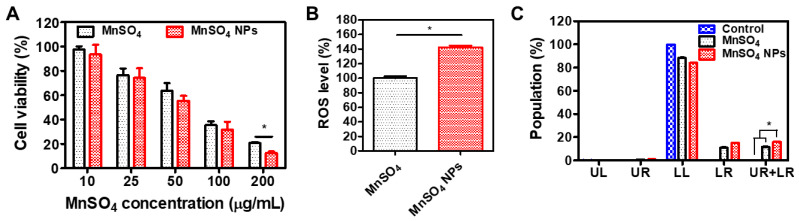
In vitro anticancer activity tests of MnSO_4_ and MnSO_4_ NPs in CT-26 cells. (**A**) Antiproliferation efficacy data of MnSO_4_ and MnSO_4_ NPs. Each point represents mean ± SD (*n* = 4). * *p* < 0.05, between two groups. (**B**) Cellular ROS assay data of MnSO_4_ and MnSO_4_ NPs. Each point represents mean ± SD (*n* = 3). * p < 0.05, between two groups. (**C**) Apoptosis induction capability of MnSO_4_ and MnSO_4_ NPs. UL: upper left; UR: upper right; LL: lower left; LR: lower right. Each point represents mean ± SD (*n* = 3). * *p* < 0.05, among indicated groups.

The anticancer potential of developed MnSO_4_ NPs was explored in CT-26 cells (Figure 8). The antiproliferation activity of designed MnSO_4_ formulations was evaluated by an MTS-based assay (Figure 8A). Both MnSO_4_ and MnSO_4_ NPs groups had concentration-dependent cytotoxicity profiles. The IC_50_ values (presented as MnSO_4_ concentration in both groups) of MnSO_4_ and MnSO_4_ NPs were 100.5 and 86.0 μg/mL, respectively. In particular, the MnSO_4_ NPs group exhibited higher cytotoxicity than MnSO_4_ group at 200 μg/mL MnSO_4_ concentration (*p* < 0.05). Compared to the micron size of MnSO_4_, MnSO_4_ NPs had colloidal size range in the aqueous media (Figure 2). A smaller particle size may have higher surface area; therefore, MnSO_4_ NPs will produce a higher OH radical amount in cancer cells rather than MnSO_4_ based on the Fenton-like reaction. Particle size reduction with HME processing and the aid of hydrophilic polymer and surfactants may support the higher cytotoxicity of MnSO_4_ NPs compared to MnSO_4_. This finding suggests the efficient antiproliferation potential of colorectal cancer cells following MnSO_4_ NPs treatment.

Cellular ROS level was investigated after applying MnSO_4_ and MnSO_4_ NPs to CT-26 cells (Figure 8B). At current tested conditions, the cellular ROS level of the MnSO_4_ NPs group was 42% higher than the MnSO_4_ group (*p* < 0.05). As revealed in the results of catalytic assays (Figure 7), Mn^2+^ ion seems to provide Fenton-like chemistry-related OH radical generation from intrinsic H_2_O_2_, which is highly accumulated in cancer cells [13,14,15]. The smaller particle size of MnSO_4_ NPs, compared to MnSO_4_, may be the reason for the enhanced ROS level in cancer cells, principally due to the improved OH radical conversion.

The apoptosis induction capability of developed NPs was also assessed by annexin V-FITC and PI staining assay (Figure 8C). The sum of percentage in the upper right (UR) and lower right (LR) panels implies the population of apoptosis after applying each treatment. In observed data (Figure 8C), both MnSO_4_ and MnSO_4_ NPs induced a higher apoptosis rate than the control (no treatment) group, meaning the apoptosis induction capacity of the Mn^2+^ ion. Notably, the population percentage of (UR + LR) panel in the MnSO_4_ NPs group was significantly higher than those of the control and MnSO_4_ groups (*p* < 0.05). An elevated cellular ROS level may lead to improved apoptosis induction efficiency, ultimately explaining the higher antiproliferation potential. All of these experimental data in CT-26 cells may indicate the application of MnSO_4_ NPs to its chemodynamic therapy.

## 4. Conclusions

Micron-sized MnSO_4_ powder was dispersed in the hydrophilic polymer (PEG 6000) and non-ionic surfactants (Span 80 and Tween 80) to make nano-sized particles after dispersing in aqueous media with the aid of HME processing. A twin-screw-installed HME system with temperature control successfully produced a homogeneous extrudate composed of MnSO_4_ and pharmaceutical excipients. Following pulverization, those HME-processed MnSO_4_ particles exhibited a nano-size diameter, spherical shape, and negative zeta potential. The proper distribution of MnSO_4_ in the hydrophilic polymer with surfactants was demonstrated by several solid-state studies. The hydroxyl radical generation activity of MnSO_4_ NPs was elucidated by catalytic assays. In colorectal cancer (CT-26) cells, Mn^2+^ ion-related Fenton-like chemistry converted intrinsic H_2_O_2_ to OH radical and elevated the cellular ROS level, apoptosis induction efficiency, and antiproliferation potential. Developed MnSO_4_ NPs may be one of the promising candidates for the chemodynamic therapy of colorectal cancer.

## Data Availability

The data presented in this study are available on request from the corresponding author.

## Supplementary Materials

The following supporting information can be downloaded at: https://www.mdpi.com/article/10.3390/pharmaceutics15071831/s1, Figure S1: XPS spectrum of C 1s in MnSO4 NPs; Figure S2: XPS spectra of O 1s in (A) MnSO_4_ and (B) MnSO_4_ NPs; Figure S3: XPS spectra of Mn 2p in (A) MnSO_4_ and (B) MnSO4 NPs; Figure S4: XPS spectra of S 2p in (A) MnSO_4_ and (B) MnSO_4_ NPs; Table S1: FT-IR spectroscopy data of MnSO4, freeze-dried mixture, and MnSO4 NPs; Table S2: XPS analytical data MnSO_4_ and MnSO_4_ NPs.
