# Supplementary material for: Manganese Sulfate Nanocomposites Fabricated by Hot-Melt Extrusion for Chemodynamic Therapy of Colorectal Cancer
Source: Pharmaceutics. 2023 Jun 27;15(7):1831. doi: 10.3390/pharmaceutics15071831 (PMC10383399; doi:10.3390/pharmaceutics15071831)
Supplement: Supplementary file 1 [file pharmaceutics-15-01831-s001.zip › pharmaceutics-2459647-supplementary.pdf]

Supplementary Information

Manganese Sulfate Nanocomposites Fabricated by Hot-Melt Extrusion for Chemodynamic Therapy of Colorectal Cancer

Da In Jeong [1,†], Sungyun Kim [1,†], Ja Seong Koo [1], Song Yi Lee [1], Minju Kim [2,3,4], Kwang Yeol Kim [2,5], Md Obyedul Kalam Azad [6,7], Mrinmoy Karmakar [1], Seongnam Chu [1,8], Byung-Jo Chae [2], Wie-Soo Kang [6] and Hyun-Jong Cho [1,*]

[1] College of Pharmacy, Kangwon National University, Chuncheon 24341, Republic of Korea; jdi0327@kangwon.ac.kr (D.I.J.), sungyun@kangwon.ac.kr (S.K.); pharmchu@dhpharm.co.kr (S.C.)
[2] Department of Animal Resources Science, College of Animal Life Sciences, Kangwon National University, Chuncheon 24341, Republic of Korea
[3] School of Animal Life Convergence Science, Hankyong National University, Anseong 17579, Republic of Korea
[4] Institute of Applied Humanimal Science, Hankyong National University, Anseong 17579, Republic of Korea
[5] Darby Genetics Inc., Anseong 17529, Republic of Korea
[6] Department of Bio-Health Technology, College of Biomedical Science, Kangwon National University, Chuncheon 24341, Republic of Korea
[7] Department of Chemistry and Biochemistry, Food and Dairy Innovation Center, Boise State University, Boise, ID 83725, USA
[8] Daehwa Pharmaceutical Co. Ltd., Seoul 06699, Republic of Korea
* Correspondence: hjcho@kangwon.ac.kr; Tel.: +82-33-250-6916
† These authors equally contributed to this work.

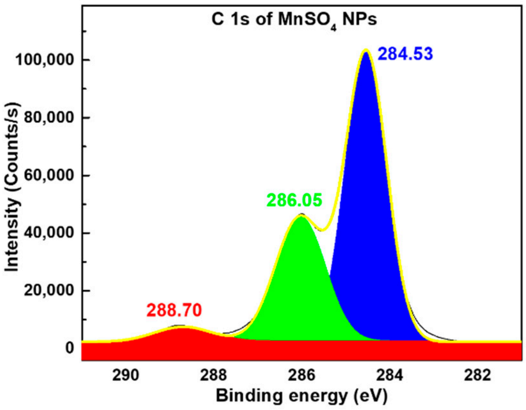

Figure S1. XPS spectrum of C 1s in $MnSO_4$ NPs.

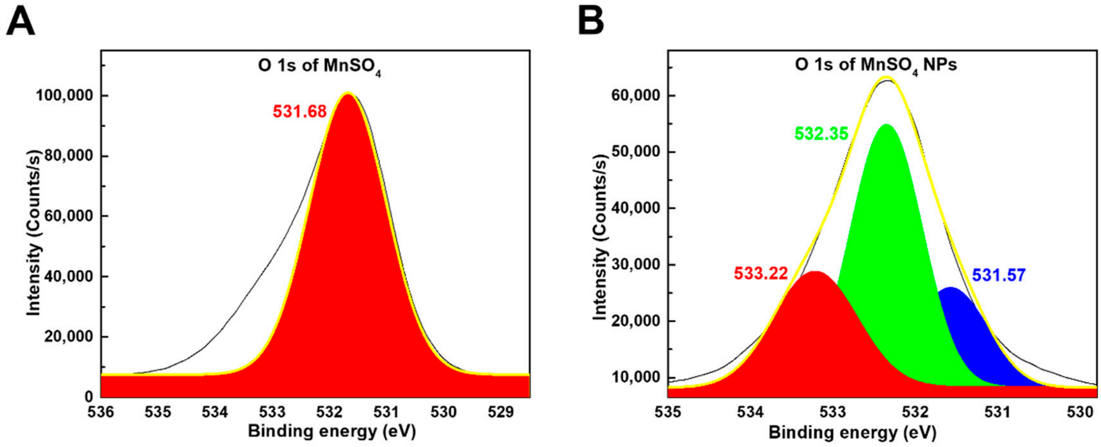

Figure S2. XPS spectra of O 1s in (A) $MnSO_4$ and (B) $MnSO_4$ NPs.

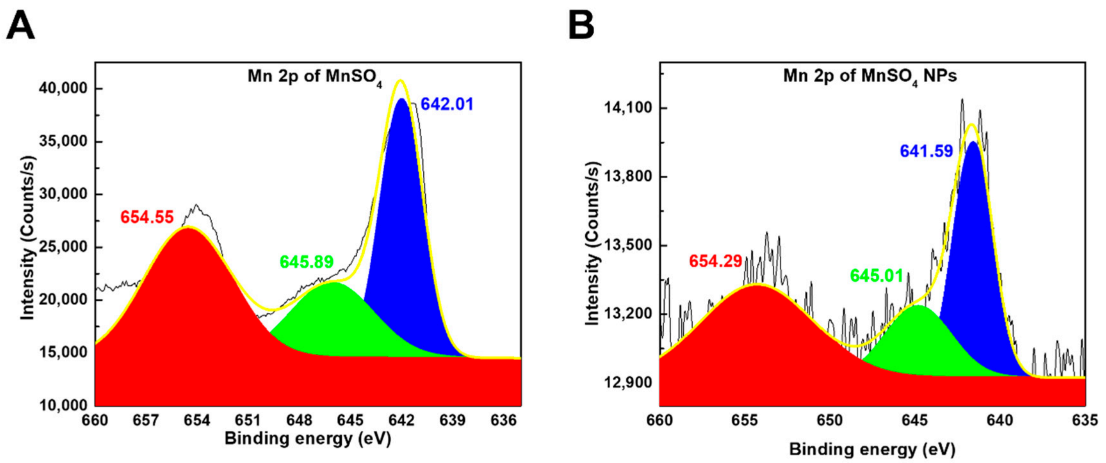

Figure S3. XPS spectra of Mn 2p in (A) $MnSO_4$ and (B) $MnSO_4$ NPs.

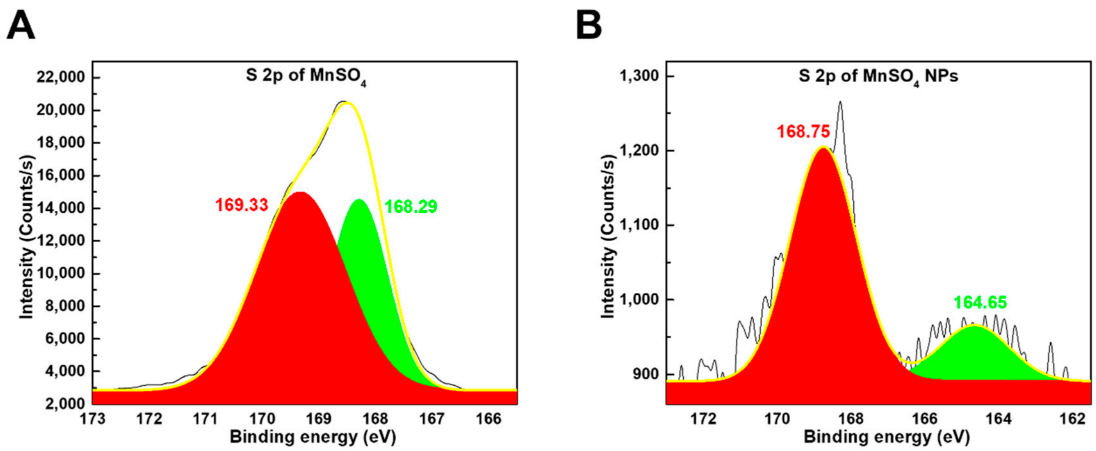

Figure S4. XPS spectra of S 2p in (A) $MnSO_4$ and (B) $MnSO_4$ NPs.

Table S1. FT-IR spectroscopy data of $MnSO_4$, freeze-dried mixture, and $MnSO_4$ NPs.

Peaks (cm^{-1})			**Explanation**
**$MnSO_4$**	**Freeze-dried mixture**	**$MnSO_4$ NPs**	
3140	3433	3411	Hydrogen-bonded O–H stretching
Absent	2882	2884	C–H stretching of $–CH_2–$
Absent	1962	1966	Overtone of C–H out-of-plane bending of –CH=CH–
Absent	1740	1740	>C=O stretching of ester
Absent	1466	1467	C–H deformation of $–CH_2–$
Absent	1341	1341	C–O stretching of ester
Absent	1279/1241	1279/1241	C–C twisting of $–(CH_2)_n–$
1090	1094	1096	$\nu_3 SO_4^{2-}$
1017	960	960	$\nu_1 SO_4^{2-}$
Absent	841	841	C=C bending
529/508	529/509	529/509	Mn–O

Table S2. XPS analytical data $MnSO_4$ and $MnSO_4$ NPs.

Species	Orbitals	Binding energy (eV)	Explanation
$MnSO_4$/$MnSO_4$ NPs	Mn $2p_{1/2}$	654.55/654.29	Shift by –0.26 and –0.42 eV for Mn $2p_{1/2}$ and Mn $2p_{3/2}$, respectively, inferred weak ionic interaction between Mn(II) and –(C=O)–O–/>CH–OH/–CH_2–OH of PEG 6000, Span 80, and Tween 80.
	Mn $2p_{3/2}$	642.01/641.59	
	Mn 2p satellite	645.89/645.01	Satellite peak of Mn(II)
	O 1s	531.68/531.57	Peak of S**O**$_4^{2-}$, shifting by –0.11 eV indicated higher electron density on S–O bond, directly connected with Mn(II). The increase in electron density on Mn(II) occurred because of weak ionic bonding with –(C=O)–O–/>CH–OH/–CH_2–OH of PEG 6000, Span 80, and Tween 80.
		Absent/532.35	–(C=**O**)–O– of PEG 6000, Span 80, and Tween 80
		Absent/533.22	>CH–**O**H/–CH_2–**O**H of PEG 6000, Span 80, and Tween 80
	C 1s	Absent/284.53	–**C**H_2–/–**C**H=**C**H– of PEG 6000, Span 80, and Tween 80
		Absent/286.05	>**C**H–OH/–**C**H_2–OH of PEG 6000, Span 80, and Tween 80
		Absent/288.70	–(**C**=O)–O– of PEG 6000, Span 80, and Tween 80
	S 2p	168.29/164.65	**S**=O of SO_4^{2-}
		169.33/168.75	**S**–O of SO_4^{2-}
